# The mediating effect of resilience and self-efficacy between physical activity and wellbeing: a meta-analytic structural equation modeling

**DOI:** 10.3389/fpsyg.2025.1621100

**Published:** 2025-08-07

**Authors:** Zhibo Cui, Zhihua Li, Tong Wang, Kaixin Li, Haoyu Zheng, Chengbo Yang

**Affiliations:** School of Sport and Training, Chengdu Sport University, Chengdu, China

**Keywords:** physical activity, wellbeing, self-efficacy, resilience, meta-analysis, structural equation modeling

## Abstract

**Background:**

While the positive relationship between physical activity and wellbeing is well-established, the mediating roles of self-efficacy and resilience remain insufficiently understood. This study employed a meta-analytic structural equation modeling (MASEM) approach to investigate whether these psychological factors mediate the relationship between physical activity and wellbeing, aiming to clarify the underlying mechanisms that contribute to this association.

**Methods:**

To achieve this, we systematically searched five databases, Web of Science, PsycINFO, SportDiscus, PubMed, and CNKI, without restrictions on language or publication date. Relevant correlation coefficients were extracted from eligible studies. A meta-analysis was conducted to evaluate the direct relationship between physical activity and wellbeing, followed by a two-stage MASEM to assess the mediating effects of self-efficacy and resilience.

**Results:**

A total of 20 studies comprising 23,377 participants revealed a significant positive correlation between physical activity and wellbeing (*r* = 0.347, *p* < 0.001). Subgroup analyses indicated stronger associations in on-site samples (*r* = 0.384) and within Eastern cultures (*r* = 0.452). Path analysis demonstrated that physical activity directly enhanced wellbeing (unstandardized effect = 0.168, *p* < 0.001), and also had significant indirect effects via self-efficacy (standardized indirect effect = 0.196, *p* < 0.001) and resilience (standardized indirect effect = 0.068, *p* < 0.001). No significant differences were found between models of subjective wellbeing and psychological wellbeing (*p* > 0.05). These findings underscore the critical mediating roles of self-efficacy and resilience in the physical activity-wellbeing pathway and offer valuable insights for designing targeted interventions to enhance mental health outcomes through physical activity.

**Conclusion:**

Physical activity is positively associated with wellbeing, with stronger effects observed in on-site sampling and within Eastern cultural contexts. Moreover, self-efficacy and resilience serve as mediating factors in the relationship between physical activity and wellbeing.

**Systematic review registration:**

https://www.crd.york.ac.uk/PROSPERO/view/CRD420251016483.

## 1 Introduction

In recent years, the relationship between physical activity and mental health has garnered increasing scholarly attention (Holland et al., 2024). Physical activity, defined as any bodily movement produced by skeletal muscles resulting in energy expenditure (Bull et al., 2020). Has been shown to significantly improve cardiorespiratory and metabolic health while also alleviating psychological symptoms such as anxiety and depression (Myers et al., 2019; Mahindru et al., 2023; Noetel et al., 2024). Despite these well-documented benefits, the association between physical activity and the promotion of positive psychological traits remains underexplored. The World Health Organization (WHO) underscores that health extends beyond the mere absence of disease to encompass complete physical, mental, and social wellbeing (International Health C, 2002). Prompting a paradigm shift in mental health research from focusing on deficits to emphasizing individual strengths (Vella and Pai, 2019). Therefore, when investigating the nexus between physical activity and mental health, it is essential to consider not only its role in reducing psychological distress but also its capacity to foster positive psychological states.

Wellbeing, often equated with happiness, refers to an individual’s evaluation and emotional experience of life quality, encompassing both hedonic and eudaimonic dimensions (Ryan and Deci, 2001). Hedonic wellbeing centers on life satisfaction and the balance of positive and negative emotions, commonly termed subjective wellbeing (Diener et al., 2018), whereas eudaimonic wellbeing involves aspects such as autonomy, environmental mastery, personal growth, positive relationships, purpose in life, and self-acceptance (Ryff, 2023). Although these constructs are correlated, Joshanloo (2016) argues that subjective wellbeing and psychological wellbeing are empirically distinct. Therefore, it is necessary to conduct independent evaluations of specific parameter groups to explore in-depth the impact of physical activity on different dimensions of wellbeing. As a positive psychological state, wellbeing has been extensively studied in relation to physical activity, with evidence indicating that individuals engaging in regular physical exercise report significantly greater wellbeing compared to inactive counterparts (Wiese et al., 2018; Ku et al., 2016). Among children and adolescents, increased sedentary behavior is strongly associated with adverse mental health outcomes and diminished wellbeing (Rodriguez-Ayllon et al., 2019). Systematic reviews corroborate the positive relationship between physical activity and wellbeing (Zhang and Chen, 2019), while the COVID-19 pandemic has further highlighted physical activity and health behaviors as critical predictors of overall wellbeing (Campoamor-Olegario et al., 2025). Notably, the emergence of digital health technologies presents promising avenues for enhancing mental health and promoting physical activity, with research demonstrating that mobile health applications effectively motivate regular exercise and provide psychological support to alleviate stress and anxiety (Alley et al., 2024; Sousa Basto and Ferreira, 2025). This advancement underscores the vital role of digital health in integrating physical exercise with mental health interventions and fostering positive health behaviors. Within this framework, it is imperative to deepen understanding of the mediating mechanisms linking physical exercise and mental health, with studies suggesting that factors such as self-efficacy and resilience may play crucial roles in this relationship (Ekkekakis et al., 2013; Lin et al., 2024).

Recent systematic reviews and meta-analyses have consistently demonstrated the positive effects of physical activity on wellbeing, psychological resilience, and self-efficacy efficacy (Fong Yan et al., 2024; Bertollo et al., 2025; Martin Ginis, 2025; Ruiz-Ranz and Asín-Izquierdo, 2025). Nevertheless, current research is limited by several key constraints. Primarily, many theoretical frameworks adopt a narrow, singular perspective rather than a holistic approach, with numerous studies concentrating either on short-term emotional effects or long-term satisfaction outcomes, thereby overlooking the dynamic mediating processes involved (Chekroud et al., 2018), This limitation impedes a comprehensive understanding of the mechanisms through which physical activity influences wellbeing. Furthermore, although psychological factors such as self-efficacy and resilience have been investigated (Olander et al., 2013; Curtis and Windsor, 2019), their individual contributions and interactive effects remain insufficiently examined, an essential gap to address in order to formulate effective, evidence-based exercise interventions aimed at enhancing mental health.

## 2 Current study and hypotheses

### 2.1 The relationship between physical activity and wellbeing

In health psychology and behavioral science, physical activity is widely acknowledged as a crucial factor for enhancing wellbeing, with extensive research demonstrating its positive effects on mental health, life satisfaction, and positive emotions (Warburton and Bredin, 2017; Liang et al., 2021; Reyes-Molina et al., 2022). However, the impact of physical activity on wellbeing varies considerably depending on the type and intensity of the activity, as well as individual differences. For instance, Eime et al. (2013) reported that individuals engaged in team sports tend to exhibit higher wellbeing scores compared to non-participants. Additionally, randomized controlled trials have shown significant improvements in the wellbeing of older adults following an eight-week physical activity intervention (Khazaee-Pool et al., 2015). Acute aerobic exercise has been found to enhance positive mood and alleviate depressive symptoms Reed and Ones (2006), whereas strength training contributes to psychological wellbeing by increasing self-efficacy and reducing anxiety (O'Connor et al., 2010).

Additionally, non-strenuous activities such as yoga and meditation have been demonstrated to effectively reduce stress and enhance the sense of life meaning, particularly in relation to subjective wellbeing (Goyal et al., 2014). In terms of intensity, vigorous exercise appears to more effectively promote hedonic wellbeing, whereas moderate-intensity activity is more beneficial for sustaining long-term psychological wellbeing, a distinction likely linked to differences in neurotransmitter release, including endorphins (Peluso and Guerra de Andrade, 2005; De Abreu et al., 2022). Age serves as a significant moderator in this relationship, with older adults experiencing notable benefits from physical activity, such as increased life meaning and decreased sadness (Yamashita et al., 2019). Potentially due to enhanced social support, maintenance of physical function, and greater resilience. Nevertheless, research investigating the suitability of specific types and intensities of physical activity across different age groups remains scarce, underscoring the need for further inquiry into how age moderates the association between physical activity and wellbeing. Based on this analysis, the following hypothesis is proposed:

*H1: Physical activity has a positive effect on wellbeing*.

### 2.2 The mediating effect of self-efficacy and resilience

Self-efficacy, defined as an individual’s belief in their capability to achieve specific goals, plays a crucial mediating role in the relationship between physical activity and wellbeing (Bandura, 1982). A longitudinal study by McAuley et al. (2005) revealed a significant correlation between physical activity and self-efficacy, a finding further supported by Joseph et al. (2014), who identified self-efficacy as the primary mediating variable linking physical exercise to quality of life in a cohort of university students. Wang et al. (2022) quantified this relationship, reporting that the total effect of physical activity on subjective wellbeing comprised a 50% direct effect and a 17.81% indirect effect mediated through enhanced self-efficacy. Similarly, Guo et al. (2024) found that self-efficacy accounted for 37.3% of the mediation between physical activity and subjective wellbeing. However, potential multicollinearity may limit the explanatory power of such mediation models. For example, Elavsky et al. (2005) demonstrated that when physical activity influences quality of life via body self-esteem and positive emotions, only positive emotions exerted a significant direct effect. Moreover, Martínez-Alvarado et al. (2022) observed in Mexican university students that although self-efficacy was among the strongest predictors of three psychological wellbeing indicators, it explained only a modest proportion of variance, indicating limitations in its predictive capacity. Collectively, these findings suggest that while self-efficacy constitutes an important mediating factor between physical activity and wellbeing, wellbeing is likely shaped by complex, multi-level synergistic mechanisms.

Resilience, defined as the personal attribute that enables individuals to strengthen in the face of adversity, is recognized as a vital psychological resource and mediator between adversity and health outcomes (Connor and Davidson, 2003; Kumpfer, 2002). Empirical evidence supports resilience’s mediating role in the relationship between physical activity and wellbeing; for instance, Ho et al. (2015) demonstrated that physical activity significantly improved the wellbeing of adolescents in Hong Kong through enhanced resilience, highlighting its importance in stress management. Likewise, Cocozza et al. (2020) found that individuals with higher physical and mental health levels are better equipped to handle life stressors, sustain regular physical activity, and consequently bolster resilience. Yao et al. (2022) further confirmed that resilience facilitates adolescents’ ability to achieve wellbeing via physical activity under stressful conditions. Beyond stress regulation, resilience plays a critical role in social–emotional learning and mental health. Supporting this, Belaire et al. (2024) revealed that physical activity significantly improves children’s self-efficacy by strengthening social support networks, thereby promoting resilience development. These findings provide valuable insights into the mechanisms underpinning resilience formation and emphasize its essential role in children’s mental health and social adaptation.

Based on these findings, the following hypotheses are proposed:

*H2: Self-efficacy mediates the relationship between physical activity and wellbeing.*

*H3: Resilience mediates the relationship between physical activity and wellbeing.*

## 3 Methods

The study was conducted in accordance with the Meta-Analysis Reporting Standards (MARS) and the Preferred Reporting Items for Systematic Reviews and Meta-Analyses (PRISMA) guidelines (Cooper, 2020; Page et al., 2021). Additionally, the study protocol has been registered in the PROSPERO database under the registration number CRD420251016483.

### 3.1 Search strategies

Following the recommendations of Siddaway et al. (2019), a focused search strategy was employed to identify relevant literature from the inception of each database through January 2025. The databases searched included Web of Science (WOS), encompassing both the Science Citation Index (SCI) and the Social Science Citation Index (SSCI), as well as PsycINFO, SportDiscus, PubMed, and the China National Knowledge Infrastructure (CNKI). The search utilized Boolean operators with the terms (“wellbeing” OR happiness OR “positive affect” OR “life satisfaction”) AND (resilience OR resilient OR “self-efficacy” OR efficacy OR “self-efficiency”) AND (exercise OR “physical activity” OR training OR sports). No restrictions were placed on language or publication type. Furthermore, reference lists of all included studies were examined to ensure a comprehensive literature retrieval process. The complete search strategy is detailed in Supplementary Tables S1–S5.

### 3.2 Inclusion and exclusion criteria

The inclusion and exclusion criteria were established following the guidelines outlined by Bergh et al. (2016) and were further informed by the research conducted by Lin et al. (2024).

The inclusion criteria were as follows: (1) published empirical journal articles, primarily cross-sectional studies, with baseline data utilized for longitudinal studies; (2) examination of the relationship between actual physical activity and wellbeing; (3) inclusion of all three variables, specifically physical activity as the predictor, self-efficacy or resilience as the mediating variable, and wellbeing as the outcome; and (4) provision of necessary data such as sample size, reliability measures, correlation coefficients, or other convertible statistical indicators.

The exclusion criteria were as follows: (1) studies focusing on irrelevant variables or health behaviors, such as exercise motivation; (2) studies examining only a single variable; (3) studies lacking sufficient statistical information to calculate effect sizes; and (4) exclusion of review articles, case reports, qualitative studies, and ineligible publication types including editorials, letters to the editor, corrigenda, study protocols, and preprints.

### 3.3 Selection of studies and coding procedures

The coding process adhered to the guidelines established by Brown et al. (2003) and Villiger et al. (2022). The research team was led by a PhD-qualified exercise psychology expert (TW) and comprised three professionally trained graduate students (ZC, HZ, and KL). All team members conducted quality assessments and data extraction in accordance with standardized protocols. In cases where data were unavailable, the authors of the original studies were contacted to obtain the necessary information. To ensure rigor and consistency, all members received training in research methodologies. Initially, Comprehensive Meta-Analysis V3 software was employed to perform preliminary coding of all selected articles, documenting author details, publication year, and study subgroups where applicable. Subsequently, coding encompassed all relevant variables and their interrelationships, including correlation coefficients and reliability measures. The process also captured sample size, sampling methods, geographical origin of samples, and cultural factors.

**Figure 1:**
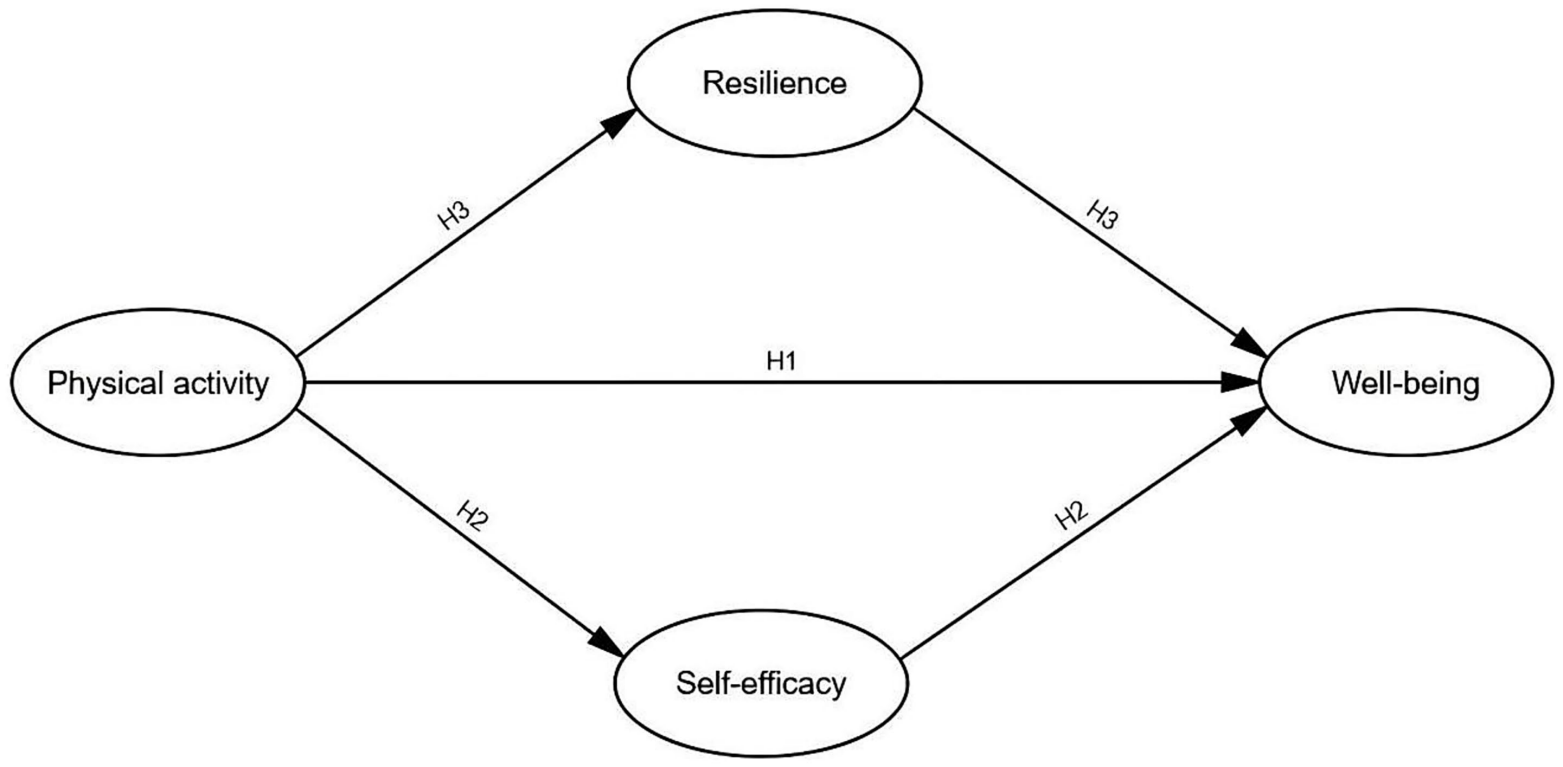
Theoretical model diagram.

Figure 1 illustrates that an initial pool of 10,020 articles from multiple databases was consolidated into a unified database using EndNote 20 software, with 3,507 duplicate records subsequently removed. Two authors (ZC and ZL) independently screened the titles and abstracts of the remaining 6,354 articles to assess their relevance to the research question, and any disagreements were resolved through consultation with a third reviewer (TW). Of the 159 articles subjected to full-text review, 8 were excluded as irrelevant (e.g., Bharti et al., 2023), 4 were conference papers (e.g., Zhu and Wang, 2023), 32 were classified as reports or reviews (e.g., Horcajo et al., 2022), 39 lacked sufficient data to calculate effect sizes (e.g., Brooks et al., 2018), and 56 were unrelated to physical activity and wellbeing (e.g., Martínez-González et al., 2021). Ultimately, 20 articles met the inclusion criteria and were selected for analysis.

#### 3.3.1 Meta-analysis

Following the Hunter and Schmidt (1990) framework, the meta-analysis was conducted using CMA 3.0 software, with effect sizes calculated via Fisher’s z-transformation. Due to the heterogeneity in populations and measurement instruments across studies, a random-effects model, as recommended by Cheung (2014), was employed, and effect sizes were reported with 95% confidence intervals to ensure statistical rigor. Pearson correlation coefficients (r) served as the primary effect size metric; for studies reporting Spearman correlations (e.g., Briki, 2018), the standard conversion formula r = 2sin(rs·*π*/6) was applied to preserve statistical equivalence (Myers and Sirois, 2006). Multidimensional correlations were integrated using the weighted average algorithm proposed by Olkin and Pratt (1958) facilitated by an online tool.^1^ Effect sizes were interpreted according to Cohen (2013), guidelines, categorizing values as small (0.10–0.30), medium (0.30–0.50), or large (0.50–1.00).

To enhance the reliability of the research findings, methodological controls were implemented from two perspectives. First, publication bias was assessed using the fail-safe number (FSN) and Egger’s test, with Rosenthal (1979) criterion suggesting that an FSN exceeding 100 studies provides sufficient evidence to rule out publication bias. Second, heterogeneity among studies was evaluated using the Q test; when the Q value was significant, subgroup analyses were conducted to identify sources of heterogeneity. Potential moderating variables included: (1) wellbeing type, categorized as subjective happiness and psychological happiness according to Joshanloo (2019); (2) age groups divided into <25 years, 25–44 years, and ≥45 years in line with (WHO, 2000); (3) survey modality, distinguishing between online and on-site data collection to account for potential limitations of online surveys regarding response rates and reliability as noted by Nulty (2008); and (4) cultural context, classified as “Eastern culture” and “Western culture” based on geographical location, following (Gaston-Breton et al., 2021; Puttevils et al., 2021), to explore differences in wellbeing outcomes.

#### 3.3.2 Structural equation modeling

Following Cheung and Chan (2005), a two-stage structural equation modeling (TSSEM) approach was employed. In the first stage, study effect sizes were integrated using the random-effects model in CMA 3.0 software, and a pooled correlation matrix was generated. Recognizing that meta-analytic studies often involve variables measured on differing scales, which can introduce methodological bias, reliability correction was applied to all study results. For studies that did not report reliability estimates, missing values were imputed using the average of available reliability coefficients from comparable studies, as recommended by Balkundi and Harrison (2006). For example, if 17 of the 20 studies included reliability estimates for wellbeing, we used the average of these reliabilities as the best estimate of the reliability of wellbeing in the remaining three studies. This is a fairly routine practice in meta-analysis (Jiang et al., 2012).

In the second phase of analysis, structural equation modeling was conducted using AMOS 27.0 software with correlation matrices as input (Jak, 2015). To account for variability in sample sizes across different variable pairs, the harmonic mean of all relevant sample sizes was calculated and applied as the effective sample size for coefficient estimation (Burke and Landis, 2013). The chi-square (χ^2^) test served as the primary measure of model fit; however, acknowledging Browne and Cudeck (1992) observation that χ^2^ may overestimate fit in large samples, the Bollen-Stine bootstrap method was employed to enhance estimation accuracy (Corrêa Ferraz et al., 2022). A comprehensive assessment of model fit was conducted using multiple indices, including χ^2^/df, the goodness-of-fit index (GFI), adjusted goodness-of-fit index (AGFI), standardized root mean square residual (SRMR), comparative fit index (CFI), incremental fit index (IFI), and Hoelter’s critical N indicator (Jackson et al., 2009).

### 3.4 Study quality assessment

The quality of the included studies was independently evaluated by two reviewers (ZC and HZ), with any disagreements resolved through consultation with a third reviewer (CY). Effect sizes were aggregated using correlation coefficients. Study quality was assessed using the 13-item Quality Assessment and Validity Tool developed by Cicolini et al. (2014), which examines research design, sample characteristics, measurement, and statistical analysis. Each item was scored as 0 (not met) or 1 to 2 (met), with total scores categorized as low quality (0–4), medium quality (5–9), and high quality (10–14). This assessment tool has been employed in previous meta-analyses, including those by Fragkos et al. (2020).

## 4 Results

### 4.1 Characteristics of the included studies

Table 1 presents a comprehensive summary of the key characteristics and outcomes of the 20 studies included in the analysis, which were conducted across diverse locations including Northern Ireland, the United States, Switzerland, Italy, and China. The combined sample comprised 23,377 participants, with individual study sample sizes ranging from 364 to 3,485. Participant ages spanned from 10 to over 68 years. In terms of sampling methodology, 12 studies (54.55%) employed face-to-face sampling, 7 studies (31.82%) utilized online sampling, and 3 studies did not specify their sampling methods and were therefore categorized as ‘not reported.’

**Table 1:** Summary of characteristics of studies included in the meta-analysis.

Study name	Sample size	Age	Sampling method	Area	Culture	Include variable	Quality assessment
Andretta and McKay (2020)	3,485	Not report	On-site	Northern Ireland	Eastern	PA, PWB, SE	Medium
Belaire et al. (2024)	534	10 ± 1.018	On-site	United States	Eastern	PA, PWB, PR	Medium
Briki (2018)	501	32.16 ± 10.43	Online	United States	Western	PA, SWB, SE	Medium
Buchecker and Degenhardt (2015)	1,200	Not report	Not report	Switzerland	Western	PA, SWB, PR	High
Cocozza et al. (2020)	1,182	55.73 ± 15.70	On-site	Italy	Western	PA, SWB, PR	Medium
Donizzetti (2023)	1,061	37.3 ± 14.13	Online	Italy	Western	PA, PWB, SE	High
Song (2020)	1794	15.35 ± 2.92	Not report	China	Eastern	PA, SWB, SE, PR	High
Guo and Jiang (2023)	364	26.7 ± 8.1	Not report	China	Eastern	PA, SWB, SE	High
Guo et al. (2024)	1,100	19.65 ± 1.1	Online	China	Eastern	PA, PWB, SE	High
Ho et al. (2015)	779	12.28 ± 0.77	On-site	China	Eastern	PA, PWB, SE, PR	Medium
Lin et al. (2022)	520	68.16 ± 2.25	On-site	China	Eastern	PA, SWB, SE	High
Xi et al. (2024)	2,311	60.79 ± 6.86	Online	China	Eastern	PA, PWB, PR	Medium
Meng et al. (2024)	746	Not report	On-site	China	Eastern	PA, SWB, SE, PR	Medium
Rejeski et al. (2001)	854	Not report	On-site	United States	Western	PA, SWB, SE	High
Van Liew et al. (2013)	363	69 ± 5.6	Not report	United States	Western	PA, WB, SE	Medium
Wang et al. (2022)	826	20.13 ± 1.05	On-site	China	Eastern	PA, SWB, SE	High
Yang and Xiang (2021)	382	Not report	On-site	China	Eastern	PA, PWB, SE	Medium
Yao et al. (2022)	1,510	Not report	On-site	China	Eastern	PA, SWB, SE	Medium
Zhang et al. (2023)	3,143	12.94 ± 1.73	Not report	China	Eastern	PA, SWB, PR	Medium
Zhou and Zhou (2022)	722	Not report	Online	China	Eastern	PA, SWB, PR	Medium

PA = physical activity; SWB = subjective wellbeing; PWB = psychological wellbeing; WB = wellbeing; PR = resilience; SE = self-efficacy.

### 4.2 Quality assessment

Study quality assessments, presented in Table 1 and Supplementary Table S6, yielded a mean score of 9 (range 7–11). Of the studies, 12 (60%) were rated as high quality, while eight (40%) were classified as moderate quality. Notable methodological limitations included the absence of prospective designs in 75% of studies, lack of probability sampling in 80%, failure to justify sample size in 75%, unreported measures to ensure participant anonymity in 85%, and absence of outlier management in 95% of the studies.

### 4.3 Meta-analysis based on correlation

**Figure 2:**
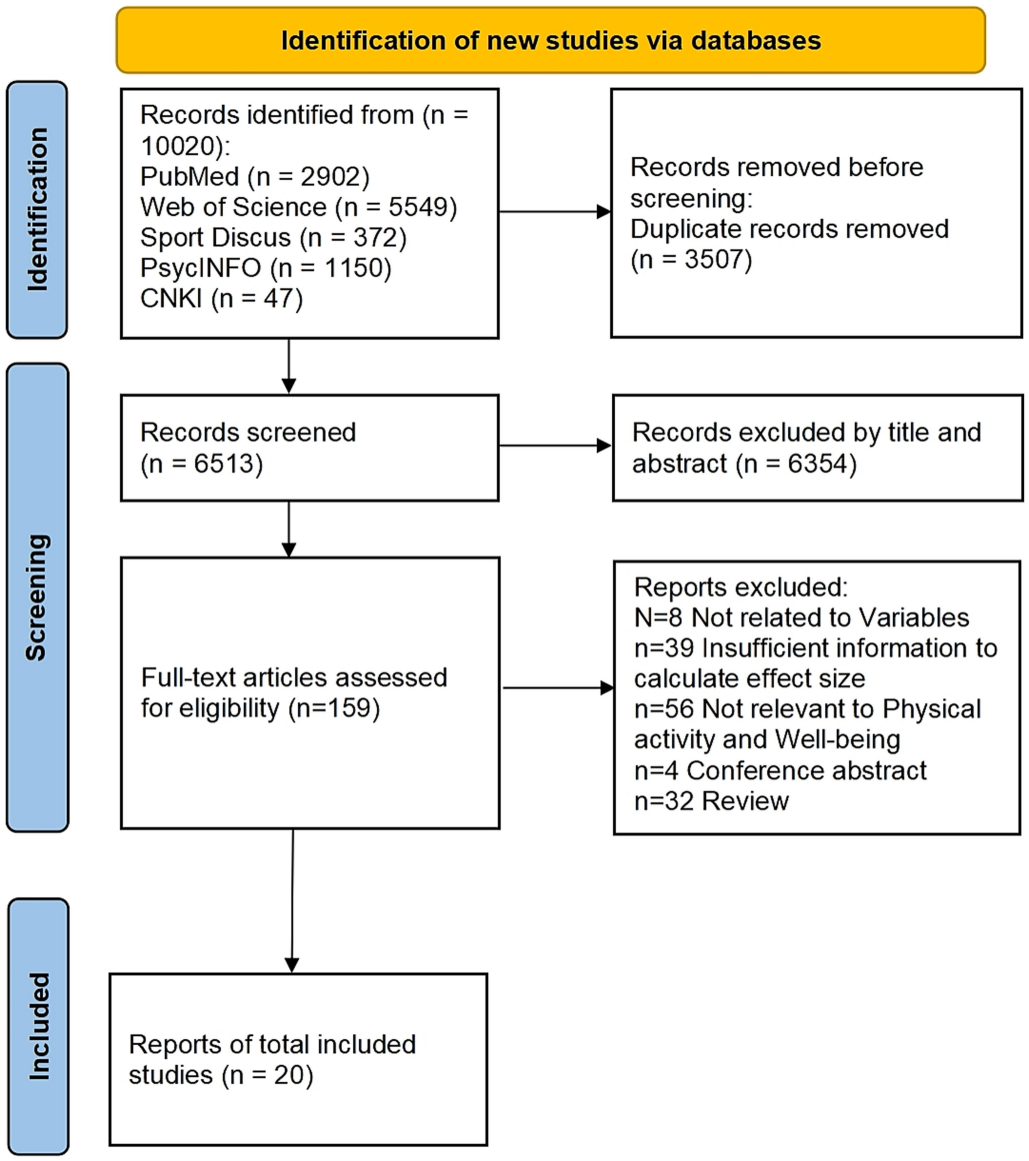
Study selection flow chart.

**Figure 3:**
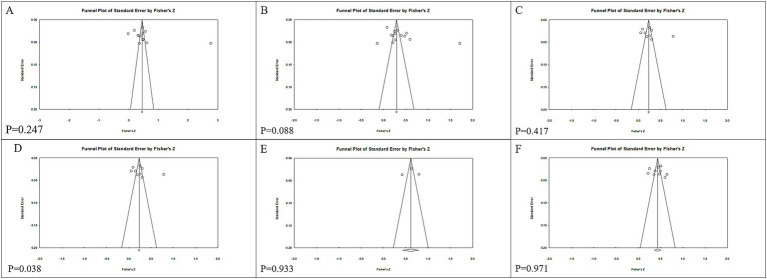
Publication bias. **(A)** SE–WB. **(B)** PA–SE. **(C)** PA–PR. **(D)** PA–WB. **(E)** PR–SE. **(F)** PR–WB.

Table 2 presents the findings of a random-effects meta-analysis estimating bivariate correlations among the variables. A significant positive association was observed between physical activity and wellbeing (*r* = 0.347, *p* < 0.001). Self-efficacy exhibited the strongest correlation with wellbeing, with an effect size of 0.516 (*p* < 0.001). The correlations between physical activity and self-efficacy (*r* = 0.363, *p* < 0.001) and between physical activity and resilience (*r* = 0.259, *p* < 0.001) were also significant. Additionally, a strong positive relationship was found between resilience and self-efficacy (*r* = 0.546, *p* < 0.001). Examination of publication bias revealed asymmetry in the funnel plot (Figure 2) for the physical activity-wellbeing relationship, corroborated by Egger’s test (*p* = 0.038). Nevertheless, the high FSN (FSN = 660, well above the threshold of 100) suggests that the results remain robust despite potential publication bias (Figure 3).

**Table 2:** Summary of bivariate correlation estimated by random-effects meta-analysis.

Pairwise relationships	*K*	*N*	*r*	95% CI		Heterogeneity	Publication bias	
				Lower	Upper	*Q*-value	Egger’s test	FSN
SE ↔ WB	13	13,431	0.516^**^	0.316	0.672	2336.219^**^	0.247	4,066
PA ↔ SE	14	14,285	0.363^**^	0.221	0.490	1106.446^**^	0.088	5,275
PA ↔ PR	9	12,411	0.259^**^	0.148	0.364	328.295^**^	0.417	1858
PA ↔ WB	20	23,377	0.347^**^	0.236	0.449	1637.799^**^	0.038	660
PR ↔ SE	3	3,319	0.546^**^	0.403	0.663	53.271^**^	0.993	934
PR ↔ WB	10	13,265	0.41^**^	0.344	0.472	175.369^**^	0.971	5,932

PA, physical activity; SWB, subjective wellbeing; PWB, psychological wellbeing; WB, wellbeing; PR, resilience; SE, self-efficacy; K, number of correlation coefficients; N, cumulative sample size; r, estimated correlation coefficient; CI, confidence interval; FSN, fail-safe number; **p < 0.001.

Given the substantial heterogeneity observed in the relationship between physical activity and wellbeing (see Table 2), subgroup analyses were conducted based on type of happiness, age, and cultural background. Results indicated that the correlation between physical activity and psychological wellbeing (*r* = 0.460, *p* = 0.001) was stronger than that between physical activity and subjective wellbeing (*r* = 0.298, *p* < 0.001), although this difference was not statistically significant (*p* > 0.05). No significant differences were found across age groups (*p* > 0.05), with the 25–44 years group exhibiting the highest correlation (*r* = 0.573, *p* = 0.160), followed by the ≥45 years group (*r* = 0.230, *p* = 0.001), and the <25 years group showing a moderate correlation (*r* = 0.334, *p* < 0.001). Sampling method significantly moderated the relationship (*Q* = 4.010, *p* < 0.05), with on-site sampling yielding a higher correlation (*r* = 0.195, *p* < 0.001) compared to online sampling. Additionally, cultural background was a significant moderator (*Q* = 9.381, *p* < 0.05), with the association between physical activity and wellbeing being more pronounced among individuals from Eastern cultures (*r* = 0.452, *p* < 0.001) than those from Western cultures (*r* = 0.171, *p* < 0.001) (Table 3).

**Table 3:** Subgroup analysis results.

Subgroup	*K*	Effect size (*r*)	*P*	95% CI		*Q*-value
				Lower	Upper	
Type						
Psychological wellbeing	7	0.460	0.001	0.198	0.661	1.359
Subjective wellbeing	12	0.298	<0.001	0.186	0.402	
Age						
25–44 years	3	0.573	0.160	−0.251	0.915	1.883
≥45 years	4	0.230	0.001	0.096	0.355	
<25 years	7	0.334	<0.001	0.205	0.451	
Sampling methods						
On-site	10	0.384	<0.001	0.229	0.521	4.010^*^
Online	5	0.195	<0.001	0.091	0.296	
Culture						
Eastern	12	0.452	<0.001	0.289	0.589	9.381^**^
Western	8	0.171	<0.001	0.106	0.236	

K = number of correlation coefficients; r = estimated correlation coefficient; *p < 0.005; **p < 0.001, *** = *p* < 0.001.

### 4.4 Meta-analytic structural equation modeling

#### 4.4.1 Path analysis

Table 4 presents the first stage of the TSSEM analysis, in which effect sizes were calculated from the correlation coefficients of 20 independent studies. The resulting correlation matrix was then imported into AMOS 27.0 software to perform the Meta-SEM analysis. Measurement error was estimated as 1 minus the average reliability coefficient (*α*), while the effective sample size was determined using the harmonic mean of the individual study sample sizes, following the approach of (Viswesvaran and Ones, 1995).

**Table 4:** Structural equation modeling matrix and total sample.

Variables	Physical activity	Wellbeing	Resilience	Self-efficacy
Physical activity	**0.817**	23,377(20)	12,411(11)	14,285(14)
Wellbeing	0.347	**0.884**	13,265(10)	13,431(13)
Resilience	0.259	0.410	**0.843**	3,319(3)
Self-efficacy	0.363	0.516	0.546	**0.851**

The bold diagonal indicates the average reliability coefficient of the variable. Values below the diagonal represent correlations from meta-analysis, while those above show the total sample size, with the number of studies in parentheses. Harmonic mean = 9309.675.

**Figure 4:**
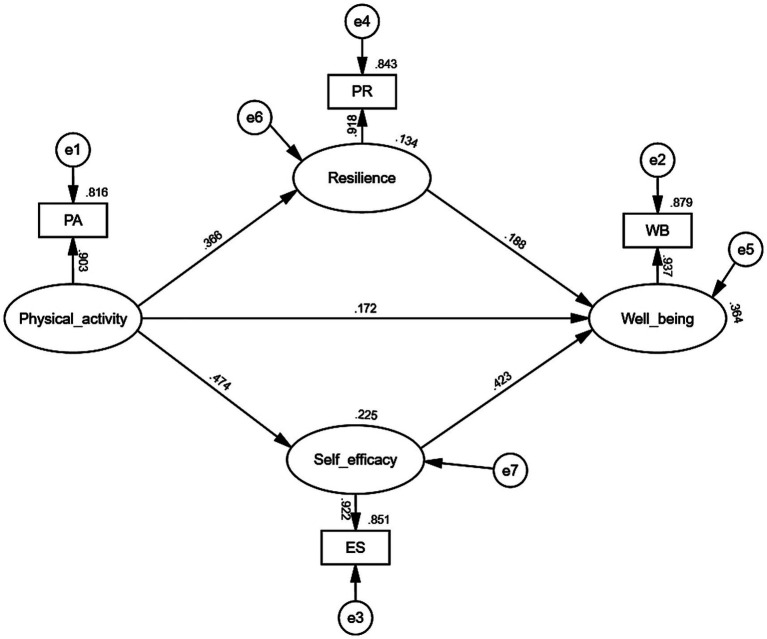
Meta-analytic structural equation model.

Table 5 and Figure 2 depict the second stage of the TSSEM analysis, where path analysis was conducted using the maximum likelihood estimation algorithm. The results indicate that physical activity significantly and positively influences multiple variables. Although physical activity’s direct effect on wellbeing is relatively modest, with a standardized coefficient of 0.172 (*p* < 0.001), this effect remains statistically significant, thus supporting hypothesis H1. The strongest path coefficient was observed for the effect of physical activity on self-efficacy, with a standardized coefficient of 0.474 (*p* < 0.001), followed by a significant effect on resilience, with a coefficient of 0.366 (*p* < 0.001). Furthermore, self-efficacy exerts a significant direct impact on wellbeing (standardized coefficient = 0.423, *p* < 0.001), while resilience also positively influences wellbeing, though to a lesser extent (standardized coefficient = 0.188, *p* < 0.001). The overall model demonstrated excellent fit, as evidenced by indices including χ^2^/df = 1.001, GFI = 1.000, AGFI = 0.999, SRMR = 0.052, CFI = 1.000, and IFI = 1.000. Additionally, Hoelter’s critical N of 10,649.157 indicates high statistical reliability and strong explanatory power of the model (Figure 4).

**Table 5:** Model path coefficient analysis with the total sample.

Path	Ustd.	S. E.	*Z*-value	*P*	Std.
PA → WB	0.168	0.013	12.915	***	0.172
PA → PR	0.368	0.012	30.769	***	0.366
PA → SE	0.476	0.012	41.227	***	0.474
SE → WB	0.412	0.012	34.866	***	0.423
PR → WB	0.183	0.011	16.705	***	0.188
Model fit: Bollen-Stine χ^2^ = 1.001, AGFI = 0.999, SRMR = 0.052, CFI = 1.000, IFI = 1.000, Hoelter’s *N* = 10649.157					

Ustd. = unstandardized coefficient, S.E. = standard error, *** = *P* < 0.001, Std. = standardized coefficient, χ^2^ = chi-square, df = degrees of freedom; GFI = goodness of fit index; AGFI = adjusted goodness of fit index; SRMR = standardized root mean square residual; CFI = comparative fit index; IFI = incremental fit index; Hoelter’s *N* = Hoelter’s critical N.

#### 4.4.2 Model comparison

**Figure 5:**
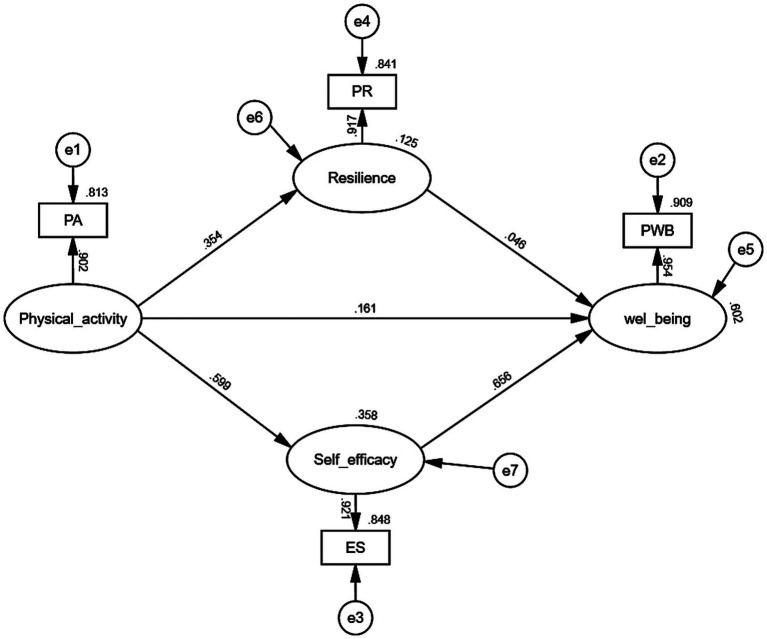
Model path coefficient analysis with psychological wellbeing.

**Figure 6:**
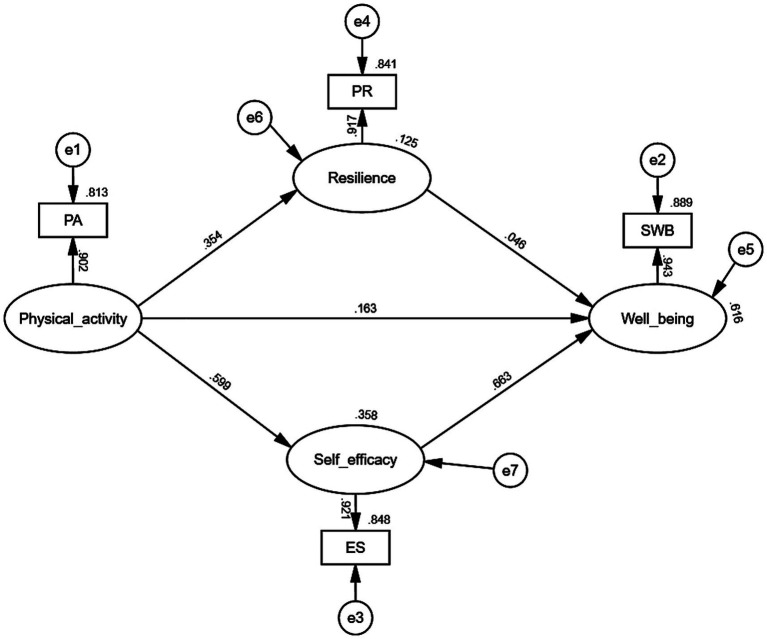
Model path coefficient analysis with subjective wellbeing.

To explore variations across studies related to wellbeing, separate MASEM analyses were conducted for samples representing subjective wellbeing (SWB) and psychological wellbeing (PWB), with the corresponding correlation matrices presented in Tables 6, 7. As shown in Table 8, all path coefficients in both models reached statistical significance. Comparative analysis indicated no significant differences between the SWB and PWB models (*p* > 0.05). Furthermore, both models demonstrated good fit, as reflected in the indices reported in Table 9 and Figures 5, 6.

**Table 6:** Structural equation modeling matrix-psychological wellbeing sample.

Variables	Physical activity	Wellbeing	Resilience	Self-efficacy
Physical activity	**0.817**	8,916(7)	3,624(3)	6,071(5)
Wellbeing	0.460	**0.884**	3,624(3)	6,071(5)
Resilience	0.212	0.462	**0.843**	779(1)
Self-efficacy	0.458	0.681	0.660	**0.851**

Harmonic = 2634.854. The bold diagonal indicates the average reliability coefficient of the variable. Values below the diagonal represent correlations from meta-analysis, while those above show the total sample size, with the number of studies in parentheses.

**Table 7:** Structural equation modeling matrix subjective wellbeing sample.

Variables	Physical activity	Wellbeing	Resilience	Self-efficacy
Physical activity	**0.817**	14,098(12)	8,787(6)	6,250(8)
Wellbeing	0.298	**0.884**	6,997(7)	6,997(7)
Resilience	0.282	0.386	**0.843**	2,540(2)
Self-efficacy	0.356	0.393	0.481	**0.851**

Harmonic = 5857.810. The bold diagonal indicates the average reliability coefficient of the variable. Values below the diagonal represent correlations from meta-analysis, while those above show the total sample size, with the number of studies in parentheses.

**Table 8:** Model path coefficient analysis with psychological wellbeing and subjective wellbeing samples.

Path	Model	Ustd.	S.E.	*Z*-value	*P*	Std.	Model comparison	
							CMIN	*P*
PA → WB	PWB	0.160	0.024	6.775	***	0.161	0.004	0.950
	SWB	0.162	0.016	10.102	***	0.163		
PA → PR	PWB	0.356	0.023	15.757	***	0.354	0.000	0.956
	SWB	0.356	0.015	23.493	***	0.354		
PA → SE	PWB	0.603	0.021	28.977	***	0.599	0.000	0.988
	SWB	0.603	0.014	43.205	***	0.599		
SE → WB	PWB	0.649	0.022	29.564	***	0.656	0.044	0.834
	SWB	0.656	0.015	44.080	***	0.663		
PR → WB	PWB	0.045	0.017	2.598	0.009	0.046	0.000	0.985
	SWB	0.046	0.012	3.874	***	0.046		

PWB = psychological wellbeing samples; SWB = Subjective wellbeing samples. ***p < 0.001.

**Table 9:** Model fit.

Model	Bollen-Stine χ^2^	df	AGFI	SRMR	CFI	IFI	Hoelter’s *N*
SWB	1.004	1	0.999	0.052	0.999	0.999	6680.547
PWB	0.976	1	0.999	0.052	0.999	0.999	3091.092

#### 4.4.3 Mediation analysis

Monte Carlo simulations with 500 replications are generally regarded as sufficient for obtaining precise statistical estimates (Chen et al., 2008). To assess the mediating effects of physical activity on wellbeing, we utilized 5,000 bootstrap samples with a 95% confidence interval to ensure more accurate and stable estimates. As presented in Table 7, the bootstrap results confirm that self-efficacy significantly and positively mediates the relationship between physical activity and wellbeing (standardized indirect effect = 0.196, *p* < 0.001), while resilience also exerts a significant positive mediating effect (standardized indirect effect = 0.068, *p* < 0.001). These findings support Hypotheses 2 and 3 (Table 10).

**Table 10:** Mediation effect test.

Path	Point estimate	Product 0f coefficient		Monte Carlo 5,000 time 95% CI			
				Bias-corrected		Percentile	
		SE	*Z*-value	Lower	Upper	Lower	Upper
Indirect effects							
Physical activity→Self-efficacy→Wellbeing	0.196	0.08	19.600	0.181	0.213	0.181	0.213
Physical activity→Resilience→Wellbeing	0.068	0.05	11.333	0.058	0.078	0.058	0.078
Direct effects							
Physical activity→Wellbeing	0.168	0.012	10.500	0.144	0.192	0.144	0.192
Total effects							
Physical activity→Wellbeing	0.432	0.012	28.800	0.408	0.456	0.409	0.456

## 5 Discussion

This study investigates the relationship between physical exercise and happiness, focusing on its impact on individual wellbeing through mediating mechanisms. Our findings indicate a significant positive association between physical activity and wellbeing (*p* < 0.05). Subgroup analyses revealed a stronger correlation with PWB (*r* = 0.460, *p* = 0.001) compared to SWB (*r* = 0.298, *p* < 0.001), although this difference was not statistically significant (*p* > 0.05). The association was most pronounced in the 25–44 age group (*r* = 0.573, *p* = 0.160) and comparatively weaker among individuals aged 45 and above (*r* = 0.230, *p* = 0.001). Additionally, effect sizes were larger in studies employing on-site sampling relative to those using online methods, and the relationship between physical activity and wellbeing was stronger within Eastern cultural contexts than in Western ones. We further examined the pathways through which physical activity influences wellbeing, focusing on the mediating roles of resilience and self-efficacy. All path coefficients were statistically significant, highlighting the important mediating effects of both self-efficacy and resilience, despite some inconsistencies reported in previous studies.

### 5.1 The relationship between physical activity and wellbeing

Our study identified a significant association between physical activity and wellbeing (*p* < 0.05), suggesting that physical activity positively influences wellbeing, consistent with prior research. Notably, the correlation between physical activity and PWB (*r* = 0.460) was stronger than that with SWB (*r* = 0.298), underscoring the multifaceted impact of physical activity on psychological dimensions of wellbeing. This aligns with findings by Hassmén et al. (2000), who reported that aerobic exercise significantly reduced depressive symptoms and enhanced SWB (*r* = 0.41, *p* < 0.01). Moreover, a meta-analysis by Buecker et al. (2021) corroborated these results, confirming a comparable association between physical activity and SWB in healthy populations.

Our findings demonstrate a significant positive association between physical activity and wellbeing (*p* < 0.05), with physical activity serving as a predictor of wellbeing. Comparative analyses revealed no significant difference between the path coefficients linking physical activity to PWB and SWB (*p* > 0.05). In contrast to earlier systematic (Marquez et al., 2020) and narrative reviews (Fox, 1999), our study conceptualizes wellbeing as comprising two distinct types: PWB and SWB. Prior research suggests that SWB is more sensitive to emotional fluctuations, whereas PWB depends on the development of more stable skills and capacities, leading to greater stability over time (Diener, 2014; Steger, 2016). Although we observed no statistically significant difference between these types (*p* > 0.05), this distinction provides a nuanced theoretical framework for understanding how physical activity differentially influences various dimensions of wellbeing. Supporting this, Vallance et al. (2023) found that higher moderate-to-vigorous physical activity levels were linked to reduced depressive symptoms, lower depression risk, and enhanced wellbeing and life satisfaction. Our study corroborates these findings through structural model validation and broadens their interpretive context.

Our analysis revealed the strongest correlation between physical activity and wellbeing in the 25–44 age group (*r* = 0.573). Individuals in this life stage often face increased occupational and familial pressures, which may amplify the relationship between physical activity and health outcomes (Infurna et al., 2020). However, this correlation did not reach statistical significance (*p* = 0.160), possibly due to limited sample size or heterogeneity within the group. Reviewing three studies related to the 25–44 age group, we identified several key factors that may influence this relationship. First, Guo and Jiang (2023) examined the relationship between physical activity and mental health in teachers within high-pressure environments, emphasizing that confounding variables such as social support and work stress might weaken the direct link between physical activity and wellbeing. Second, Briki (2018) pointed out that self-control, goal progress, and self-efficacy are crucial for enhancing wellbeing, indicating that mental wellbeing is influenced not only by physical activity but also by individual characteristics and life circumstances, complicating the research findings. Finally, Donizzetti (2023) found that the relationship between individual mental health and physical activity changed significantly during the early and later stages of the COVID-19 pandemic; the pandemic environment may affect the efficacy of physical activity, highlighting the importance of time factors in mental health. Therefore, while a positive correlation between physical activity and wellbeing was observed in the 25–44 age group, this non-significant result may reflect the complex psychosocial backdrop and various confounding factors at play. Significant differences exist between Eastern and Western cultures regarding the relationship between physical exercise and wellbeing (*p* < 0.05). Research on leisure-time physical activity among university students in Mediterranean cultures supports this, underscoring the critical role of cultural factors in shaping individuals’ experiences with physical activity (Molina-García et al., 2011). Richards et al. (2015) identified a positive dose–response relationship between physical exercise and wellbeing across multiple countries, suggesting that the type and cultural context of exercise influence its impact on wellbeing uniquely. Meta-analytic results reveal a stronger correlation in Eastern cultures (*r* = 0.452) compared to Western cultures (*r* = 0.171). Cultural background profoundly shapes cognition, communication, decision-making, and social behaviors, which likely contributes to these differences. Cross-cultural studies have documented substantial variations in the subjective experience of wellbeing, with Western cultures typically emphasizing individualism, autonomy, and self-actualization, while Eastern cultures prioritize self-transcendence and harmonious coexistence with the collective and cosmos (Joshanloo, 2014; Veenhoven, 2009). In Eastern contexts, collectivist values may amplify the mental health benefits of physical activity by fostering social bonding through group-based exercise (Lin et al., 2022). Therefore, recognizing cultural factors is essential for a comprehensive understanding of how physical activity relates to wellbeing across diverse populations, particularly in appreciating the specific influences of cultural values.

From a sampling methodology perspective, effect sizes derived from on-site sampling were larger than those obtained through online methods, suggesting that the data collection environment may systematically influence participants’ self-reported behaviors. While Hawker (2012) employed on-site measurements among nursing students, the study did not compare sampling approaches, limiting insights into how different methods may affect results. Meanwhile, online sampling has gained prominence during global health crises such as the COVID-19 pandemic. Abdelbasset et al. (2021) reported significant shifts in physical activity patterns during the pandemic, and De Man et al. (2021) observed a rapid transition from traditional on-site sampling to online survey methods using rating scales. These developments underscore the considerable impact of sampling methodology on the reliability and generalizability of research findings, highlighting the need for careful methodological consideration in study design.

### 5.2 The mediating mechanism of self-efficacy and resilience

Research indicates that exploring mediating factors in the relationship between physical exercise and mental health provides robust evidence particularly in the domains of wellbeing, self-efficacy, and resilience (White et al., 2024). This study corroborates that the positive impact of physical exercise on individual wellbeing is largely mediated through enhancements in self-efficacy and resilience. Within the self-efficacy pathway, grounded in the ‘mastery experience’ concept from social cognitive theory, engaging in progressively challenging exercise tasks allows individuals to build and strengthen their belief in their ability to overcome difficulties through practical accomplishment (Beauchamp et al., 2019). For instance, Liu et al. (2024) found that structured yoga programs focusing on systematic postural training improved female university students’ body control perception, which in turn helped regulate their emotions. Similarly, Sui et al. (2021) demonstrated that self-efficacy served as a critical mediator in managing negative emotions among healthcare workers during the COVID-19 pandemic. These findings collectively underscore the integral role of self-efficacy in mental health and highlight how physical exercise fosters both resilience and overall personal wellbeing.

The mediating role of resilience indicates that physical activity contributes to wellbeing through both direct and indirect mechanisms by enhancing resilience. This finding is consistent with the resilience model, which proposes that physical activity improves physiological functioning and influences psychological adaptation patterns through neuroplasticity (Connor and Davidson, 2003). Specifically, physical activity reduces emotional stress via physiological mechanisms, such as the release of endorphins, and strengthens individuals’ ability to cope with adversity through psychological processes, such as building frustration tolerance during exercise (Wang et al., 2023; Dong and Lin, 2025). The mediating effect size for self-efficacy (0.196) was significantly greater than that of physical activity (0.068), likely due to self-efficacy’s more direct influence on behavioral motivation. According to social cognitive theory, individuals with higher self-efficacy are more likely to adopt adaptive coping strategies, set realistic goals, and persist in the face of challenges (Benight and Bandura, 2004). Empirical studies support this view, showing that individuals with high self-efficacy are more likely to follow through on their plans and achieve goals, such as maintaining a low glycemic index diet (Miller et al., 2012). In contrast, resilience pertains to an individual’s dynamic ability to adapt to significant adversity (Luthar et al., 2000). People may display varying levels of resilience depending on the situation, sometimes showing resilience, and other times not. This context-dependent nature may explain why resilience demonstrates a smaller mediating effect compared to self-efficacy (Vella and Pai, 2019).

### 5.3 Implications of the research

The study’s results reveal a significant correlation between physical activity and wellbeing, with resilience and self-efficacy identified as key mediators, carrying important implications for clinical practice, education, and workplace interventions. In clinical settings, healthcare providers can utilize these findings to design targeted exercise programs for patients experiencing anxiety and depression, encouraging engagement in team sports or fitness activities to enhance physical health and strengthen self-efficacy. Within educational contexts, teachers can develop curricula that incorporate physical activity to improve students’ mental health by alleviating anxiety and depression while fostering self-esteem and social connectedness. In the workplace, organizations should acknowledge the mental health benefits of physical activity by implementing initiatives such as light resistance training and walking programs to promote employee wellbeing. Furthermore, policymakers must prioritize physical activity within public health strategies, recognizing its positive impact on mental health across diverse cultural contexts. Consequently, governments and organizations should intensify public health campaigns to encourage greater physical activity participation among all populations, particularly in response to the escalating prevalence of mental health challenges.

### 5.4 Limitations

This review systematically searched five Chinese and English databases and incorporated more relevant studies using an extensive screening strategy. Multiple tests for publication bias confirmed the robustness of the findings, indicating a high level of confidence in this study. However, there are notable limitations. First, although the sample covers multiple countries and regions, the uneven distribution of Eastern and Western cultures (with more studies from the East than the West) and the small sample size of older adults limit the statistical validity of the results. Therefore, future research should expand the sample size, particularly by including more participants with Western cultural backgrounds, to further investigate the generalizability of the results. Second, while existing studies have explored the relationship between physical exercise and mental health from various perspectives, the effects of potential moderating variables such as gender, baseline health status, and socioeconomic status have not been adequately analyzed. Thus, future research should further investigate the roles of these moderating variables. Additionally, most of the studies included in MASEM were cross-sectional; future research should consider updating statistical methods and incorporating more intervention studies to enhance reliability.

## 6 Conclusion

This study employed the MASEM approach to develop and test a comprehensive model examining the relationships among physical activity, wellbeing, self-efficacy, and resilience, with the goal of aiding health promoters in understanding how physical activity impacts wellbeing. The findings demonstrated a significant positive correlation between physical activity and wellbeing, with this association being particularly pronounced within Eastern cultural contexts. Both the SWB and PWB models revealed significant path relationships, with no statistically significant differences observed between them. Moreover, physical activity was found to enhance wellbeing indirectly through the mediating effects of self-efficacy and resilience, highlighting the importance of these psychological mechanisms in the physical activity-wellbeing nexus.

## Data Availability

The original contributions presented in the study are included in the article/Supplementary material, further inquiries can be directed to the corresponding author/s.

## Footnotes

^1^ https://www.psychometrica.de/correlation.html
