# Supplementary material for: The mediating effect of resilience and self-efficacy between physical activity and wellbeing: a meta-analytic structural equation modeling
Source: Front Psychol. 2025 Aug 7;16:1621100. doi: 10.3389/fpsyg.2025.1621100 (PMC12372532; doi:10.3389/fpsyg.2025.1621100)
Supplement: Supplementary file 1 [file Supplementary_file_1.docx]

Supplementary Material

[Search strategy 1](#_Toc505)

[Research quality assessment 3](#_Toc29621)

[Forest plot 6](#_Toc31557)

[Publication bias 11](#_Toc10105)

**Search strategy**

Table S1**.** Web of Science Search Results

|  | Search Query | Results |
| --- | --- | --- |
| 1 | (((AB=(physical activit*)) OR AB=(exercis*)) OR AB=(sport*)) OR AB=(training) Editions: WOS.SCI,WOS.SSCI | 1245891 |
| 2 | ((((ALL=("well-being")) OR ALL=("satisfaction")) OR ALL=("life satisfaction")) OR ALL=("positive affect")) OR ALL=(happiness) Editions: WOS.SCI,WOS.SSCI | 421179 |
| 3 | ((((ALL=("self-efficacy")) OR ALL=("self-efficiency")) OR ALL=(efficacy)) OR ALL=(resilience)) OR ALL=(resilient) Editions: WOS.SCI,WOS.SSCI | 1380125 |
| 4 | #3 AND #2 AND #1 Editions: WOS.SCI,WOS.SSCI | 5549 |

January 5, 2025 11:42:12

Table**.** S2 PsycINFO Search Results

|  | Search Query | Results |
| --- | --- | --- |
| 1 | (Abstract: "well-being" OR Abstract: happiness OR Abstract: "positive affect" OR Abstract: "life satisfaction") AND (Abstract: resilience OR Abstract: resilient OR Abstract: "self-efficacy" OR Abstract: efficacy OR Abstract: "self-efficiency") AND (Abstract: exercis# OR Abstract: physical activit# OR Abstract: training OR Abstract: sport#) | 1150 |

January 6, 2025 12:07:43 PM

Table S3**.** SPORTDiscus Search Results

|  | Search Query | Results |
| --- | --- | --- |
| 1 | AB ("well-being" or happiness or "positive affect" or "life satisfaction") AND AB (resilience or resilient or "self-efficacy" or efficacy or "self-efficiency") AND AB (exercis# or physical activit# or training or sport#) | 372 |

January 5, 2025,11:56:43 PM

Table S4**.** PubMed Search Results (N = 3,020)

|  | Search Details | Results |
| --- | --- | --- |
| 1 | "exercis*"[Title/Abstract] OR "physical activit*"[Title/Abstract] OR "sport*"[Title/Abstract] OR "training"[Title/Abstract] | 1,167,385 |
| 2 | ((("well-being"[Title/Abstract]) OR (happiness[Title/Abstract])) OR ("positive affect"[Title/Abstract])) OR ("life satisfaction"[Title/Abstract]) | 166,207 |
| 3 | (((("self-efficacy"[Title/Abstract]) OR ("self-efficiency"[Title/Abstract])) OR (efficacy[Title/Abstract])) OR (resilience[Title/Abstract])) OR (resilient[Title/Abstract]) | 1,282,782 |
| 4 | (("exercis*"[Title/Abstract] OR "physical activit*"[Title/Abstract] OR "sport*"[Title/Abstract] OR "training"[Title/Abstract]) AND (((("well-being"[Title/Abstract]) OR (happiness[Title/Abstract])) OR ("positive affect"[Title/Abstract])) OR ("life satisfaction"[Title/Abstract]))) AND ((((("self-efficacy"[Title/Abstract]) OR ("self-efficiency"[Title/Abstract])) OR (efficacy[Title/Abstract])) OR (resilience[Title/Abstract])) OR (resilient[Title/Abstract])) | 2,902 |

January 5, 2025 10:35:19

Table S5**.** CNKI Search Results (N = 47)

|  | Search Details | Results |
| --- | --- | --- |
| 1 | (身体活动 + 运动 + 锻炼) AND (幸福感 + 生活满意度 + 积极情绪) AND (抗逆力 + 心理韧性 + 自我效能感 + 自我效能) | 47 |

January 5, 2025, 13:52:53

**Research quality assessment**

**Table S6 Quality Assessment of Included Studies**

| ID | Prospective study | Probability sampling | Sample size justification | Multi-site sampling | Protection of anonymity | Response rate ≥ 60% | Reliable independent variable measurement | Valid instrument used for independent variable measurement | Valid instrument used for dependent variable measurement | Internal consistency ≥ 0.70 for dependent variable scale (if used) | Use of a theoretical framework | Analysis of correlations for multiple outcomes | Management of outliers | Total | Classification of quality |
| --- | --- | --- | --- | --- | --- | --- | --- | --- | --- | --- | --- | --- | --- | --- | --- |
| Andretta and McKay (2020) | 0 | 0 | 0 | 1 | 0 | 0 | 1 | 1 | 1 | 2 | 1 | 1 | 0 | 8 | Medium |
| Belaire et al. (2024) | 0 | 0 | 0 | 1 | 0 | 1 | 1 | 1 | 1 | 2 | 1 | 1 | 0 | 9 | Medium |
| Briki (2018) | 0 | 0 | 0 | 0 | 0 | 1 | 1 | 1 | 1 | 2 | 1 | 1 | 0 | 8 | Medium |
| Buchecker and Degenhardt (2015) | 0 | 0 | 1 | 1 | 0 | 1 | 1 | 1 | 1 | 2 | 1 | 1 | 0 | 10 | High |
| Cocozza et al. (2020) | 0 | 0 | 0 | 1 | 1 | 0 | 1 | 1 | 1 | 2 | 1 | 1 | 0 | 9 | Medium |
| Donizzetti (2023) | 1 | 0 | 0 | 1 | 0 | 1 | 1 | 1 | 1 | 2 | 1 | 1 | 0 | 10 | High |
| Song (2020) | 0 | 0 | 0 | 1 | 1 | 1 | 1 | 1 | 1 | 2 | 1 | 1 | 0 | 10 | High |
| Guo. and Jiang (2023) | 0 | 1 | 1 | 0 | 0 | 1 | 1 | 1 | 1 | 2 | 1 | 1 | 0 | 10 | High |
| Guo et al. (2024) | 0 | 1 | 0 | 1 | 0 | 1 | 1 | 1 | 1 | 2 | 1 | 1 | 0 | 10 | High |
| Ho et al. (2015) | 0 | 0 | 1 | 0 | 1 | 0 | 1 | 1 | 1 | 2 | 1 | 1 | 0 | 9 | Medium |
| Lin. et al. (2022) | 0 | 1 | 0 | 1 | 0 | 1 | 1 | 1 | 1 | 2 | 1 | 1 | 0 | 10 | High |
| Xi et al. (2024) | 0 | 0 | 0 | 1 | 0 | 1 | 1 | 1 | 1 | 2 | 1 | 1 | 0 | 9 | Medium |
| Meng et al. (2024) | 0 | 0 | 0 | 1 | 0 | 1 | 1 | 1 | 1 | 2 | 1 | 1 | 0 | 9 | Medium |
| Rejeski et al. (2001) | 0 | 0 | 1 | 1 | 0 | 1 | 1 | 1 | 1 | 2 | 1 | 1 | 0 | 10 | High |
| Van Liew et al. (2013) | 0 | 0 | 0 | 0 | 0 | 0 | 1 | 1 | 1 | 2 | 1 | 1 | 0 | 7 | Medium |
| Wang et al. (2022) | 1 | 1 | 1 | 1 | 0 | 0 | 1 | 1 | 1 | 2 | 1 | 1 | 0 | 11 | High |
| Yang (2021) | 1 | 0 | 0 | 0 | 0 | 0 | 1 | 1 | 1 | 1 | 1 | 1 | 0 | 7 | Medium |
| Yao et al. (2022) | 0 | 0 | 0 | 0 | 0 | 1 | 1 | 1 | 1 | 2 | 1 | 1 | 0 | 8 | Medium |
| Zhang et al. (2023) | 1 | 0 | 0 | 0 | 0 | 0 | 1 | 1 | 1 | 2 | 1 | 0 | 0 | 7 | Medium |
| Zhou and Zhou (2022) | 1 | 0 | 0 | 1 | 0 | 1 | 0 |  | 1 | 2 | 1 | 1 | 1 | 9 | Medium |

**Forest plot**

**Supplementary Figure S1.** Forest plot of the correlation between PA and WB

**Supplementary Figure S2.** Forest plot of the correlation between SE and WB

**Supplementary Figure S3.** Forest plot of the correlation between PA and ES

**Supplementary Figure S4.** Forest plot of the correlation between PA and PR

**Supplementary Figure S5.** Forest plot of the correlation between PR and ES

**Supplementary Figure S6.** Forest plot of the correlation between PR and WB

**Supplementary Figure S7.** Forest plot of subgroup analysis by age

**Supplementary Figure S8.** **Forest plot of subgroup analysis by culture**

**Supplementary Figure S9.** Forest plot of subgroup analysis by sampling method (1=On-site, 2=Online)

**Publication bias**

**Supplementary Figure S10.** Publication bias Self-Efficacy ↔ Well-being (P=0.247)

**Supplementary Figure S11.** Publication bias Physical Activity ↔ Self-Efficacy (P=0.088)

**Supplementary Figure S12.** Publication bias Physical Activity ↔ Resilience (P=0.417)

**Supplementary Figure S13.** Publication bias Physical Activity ↔ Well-being (P=0.038)

**Supplementary Figure S14.** Publication bias Resilience ↔ Self-Efficacy (P=0.933)

**Supplementary Figure S15.** Publication bias Resilience ↔ Well-being (P=0.971)
